# Activation of SAPK/JNK by camptothecin sensitizes androgen-independent prostate cancer cells to Fas-induced apoptosis

**DOI:** 10.1054/bjoc.2000.1149

**Published:** 2000-06

**Authors:** A P Costa-Pereira, S L McKenna, T G Cotter

**Affiliations:** Tumour Biology Laboratory, Department of Biochemistry, University College, Ireland

**Keywords:** prostate cancer, apoptosis, Fas, camptothecin, SAPK/JNK

## Abstract

We have previously shown that the androgen-independent prostate cancer cells DU145, despite expressing Fas and FasL, were resistant to anti-Fas-induced apoptosis, and that this resistance could be overcome by pretreating the cells with sublethal doses of camptothecin. Here, we provide evidence that SAPK/JNK activity is required for camptothecin sensitization to anti-Fas-induced apoptosis. Camptothecin, but not Fas ligation, was shown to activate SAPK/JNK in a time-dependent manner, and to induce c-Jun expression. The effects were more prominent in cells treated with both camptothecin and anti-Fas. The expression levels of MKP-1, a phosphatase which regulates SAPK/JNK and which has been implicated in prostate cancer resistance to apoptosis, remained unchanged. Inhibition of caspases had no effect on the SAPK/JNK activation, suggesting that this activation is an upstream event in the Fas-signalling pathway, and is independent of caspase activity. Antisense oligonucleotides targeted to JNK1 and JNK2 reversed the effect of camptothecin. These results suggest that stress kinase activation can significantly influence the fate of androgen-independent prostate cancer cells following Fas receptor ligation. © 2000 Cancer Research Campaign


British Journal of Cancer (2000) **82**(11), 1827–1834
© 2000 Cancer Research Campaign
DOI: 10.1054/ bjoc.2000.1149, available online at http://www.idealibrary.com on IDEAL®

### Activation of SAPK/JNK by camptothecin sensitizes androgen-independent prostate cancer cells to Fas-induced apoptosis

**AP Costa-Pereira*, SL McKenna† and TG Cotter**

Tumour Biology Laboratory, Department of Biochemistry, University College, Ireland

**Summary** We have previously shown that the androgen-independent prostate cancer cells DU145, despite expressing Fas and FasL, were resistant to anti-Fas-induced apoptosis, and that this resistance could be overcome by pretreating the cells with sublethal doses of camptothecin. Here, we provide evidence that SAPK/JNK activity is required for camptothecin sensitization to anti-Fas-induced apoptosis. Camptothecin, but not Fas ligation, was shown to activate SAPK/JNK in a time-dependent manner, and to induce c-Jun expression. The effects were more prominent in cells treated with both camptothecin and anti-Fas. The expression levels of MKP-1, a phosphatase which regulates SAPK/JNK and which has been implicated in prostate cancer resistance to apoptosis, remained unchanged. Inhibition of caspases had no effect on the SAPK/JNK activation, suggesting that this activation is an upstream event in the Fas-signalling pathway, and is independent of caspase activity. Antisense oligonucleotides targeted to JNK1 and JNK2 reversed the effect of camptothecin. These results suggest that stress kinase activation can significantly influence the fate of androgen-independent prostate cancer cells following Fas receptor ligation. © 2000 Cancer Research Campaign

**Keywords**: prostate cancer; apoptosis; Fas; camptothecin; SAPK/JNK

Novel topoisomerase I inhibitors are being developed as potential therapeutic agents for a variety of solid tumours (de Souza et al, 1997). The topoisomerase I inhibitor camptothecin has been shown to have substantial activity against a variety of prostate cancer cell lines in vitro. Recently, 9-aminocamptothecin, a derivative of camptothecin, has been shown to be active against hormone-refractory prostate cancer cells, and it is being tested in a phase II clinical trial (de Souza et al, 1997). Camptothecin has been shown to induce single-strand DNA breaks in the presence of topoisomerase I, which is the major target for its anti-tumour effect (Hsiang et al, 1989).

The cell surface receptor Fas (CD95/APO-1) belongs to the nerve growth factor/tumour necrosis factor (NGF/TNF) receptor family, and its main function is believed to be the induction of apoptosis in a variety of cell types (Yonehara et al, 1989; Oehm et al, 1992). The Fas/FasL system plays a key role in the development, homeostasis, modulation and function of the immune system (Leibson, 1997). In addition, Fas has been implicated in the maintenance of immune privilege in tissues such as the brain, eyes and testis, presumably by inducing apoptosis in infiltrating inflammatory cells (Griffith et al, 1995). The concept of a ‘Fas counter-attack’ has emerged as a possible mechanism used by tumours to escape immune surveillance (O’Connell et al, 1996; Villunger et al, 1997). Recently, it has been shown that the normal epithelium, as well as locally invasive prostate cancers, have the potential to undergo Fas-induced apoptosis, whereas metastatic prostate cancers have a reduced susceptibility to Fas-mediated apoptosis (Hedlund et al, 1998; Costa-Pereira and Cotter, 1999).

DNA-damaging agents and DNA-reactive chemicals are able to induce several cellular responses, the most general one being stabilization of p53 and subsequent $G_0/G_1$ cell cycle arrest (Kastan et al, 1991; Kuerbitz et al, 1992). However, many cancers express mutant p53. Thus, additional responses must be used by cytotoxic agents to induce apoptosis. Some DNA-damaging agents also induce the activation of stress-activated protein kinases, such as the SAPK/JNK and/or p38MAPK cascades (Dhanasekaran and Reddy, 1998; Sánchez-Perez et al, 1998). The SAPK/JNK-signalling pathway, which is activated in response to a variety of cytotoxic stresses, involves the sequential phosphorylation of MEKK, MKK4 and/or MKK7, and SAPK/JNK (Kyriakis and Avruch, 1996). MKK7 has been shown to be induced by pro-inflammatory cytokines and by osmotic stress (Finch et al, 1997). Interestingly, Fas-induced apoptosis in T-cells was shown to involve MKK7-SAPK/JNK- and MKK6-p38MAPK-signalling modules (Toyoshima et al, 1997). It appears that pro-inflammatory cytokines preferentially activate MKK7, whereas other stresses activate both MKK4 and MKK7 (Dhanasekaran and Reddy, 1998). Thus, MKK4 and MKK7 seem to be differentially regulated and it raises an interesting possibility that different stimuli recruit MKK4 and MKK7 based on their need to co-stimulate the p38MAPK cascade or not. Phosphorylated and activated SAPK/JNK translocates into the nucleus where it can phosphorylate a number of transcription factors, such as c-Jun and ATF-2 (Gupta et al, 1995).

*Received 26 July 1999*
*Revised 2 February 2000*
*Accepted 5 February 2000*

*Correspondence to:* TG Cotter

*Present address: Biochemical Regulatory Mechanisms, Imperial Cancer Research Fund, 44 Lincoln’s Inn Fields, London WC2A 3PX, UK
†Present address: Signal Transduction Laboratory, Department of Biochemistry, University College, Ireland

The importance of SAPK/JNK activity in Fas-signalling pathways is still controversial, with evidence for and against its involvement (Goillot et al, 1997; Juo et al, 1997; Lenczowski et al, 1997). Although DU145 cells are resistant to anti-Fas-mediated cytotoxicity, the cells retain the cellular components of the apoptotic machinery necessary to undergo death triggered by Fas, as the resistance can be overcome by pretreating the cells with sublethal doses of camptothecin. This effect is independent of receptor expression, as levels were the same in resistant and sensitized cells (Costa-Pereira and Cotter, 1999). Other cytotoxic drugs (e.g. topoisomerase II inhibitors, non-classical alkylating agents, RNA transcription inhibitors) have also been shown to sensitize DU145 cells, although to a lesser extent (Uslu et al, 1997; Costa-Pereira and Cotter, 1999). Since different modes of action sensitize DU145 cells to anti-Fas-induced apoptosis, a common 'stress-activated pathway' is likely to be targeted. Here, we provide evidence that camptothecin, but not anti-Fas, strongly and persistently activated SAPK/JNK in DU145 cells. Furthermore, we show that activation of SAPK/JNK, and induction of c-Jun expression correlate with apoptosis. Antisense oligonucleotides targeted to JNK1 and JNK2 reversed camptothecin sensitization to anti-Fas-mediated apoptosis, suggesting a key role for SAPK/JNK in Fas-mediated apoptotic cell death in DU145 cells.

### MATERIALS AND METHODS

### Cell lines and reagents

The metastatic prostate cancer cell line DU145 was obtained from American Type Culture Collection (Rockville, MD, USA). Primary antibodies used in this study were anti-Fas IgM, clone Ch-11 (Upstate Biotechnology, Waltham, MA, USA); anti-JNK1, C-17 (Santa Cruz); anti-c-Jun/AP-1, Ab-1 (Oncogene Research Products, MA, USA); and anti-β-actin (Sigma, Poole, UK). The caspase inhibitor benzyloxycarbonyl-Val-Ala-Asp-fluoromethyl ketone (z-VAD) was purchased from Enzyme System Products (Livermore, CA, USA). Cell culture reagents were purchased from Gibco-BRL (Paisley, UK), protein gel electrophoresis reagents from BioRad (Hertfordshire, UK) and the SAPK/JNK assay kit from New England BioLabs (UK) Ltd. (Hertfordshire, UK). The phosphorothioate oligonucleotides used in this study were purchased from Genosys (UK). Cytotoxic drugs were from Sigma, and all other reagents were obtained from BDH (Cork, Ireland).

### Cell culture

Cells were cultured in RPMI-1640 medium containing 5% (v/v) fetal calf serum, 2 mM L-glutamine, supplemented with penicillin–streptomycin (10 IU ml$^{-1}$, 10 µg ml$^{-1}$) and fungizone amphotericin B (2.5 µg ml$^{-1}$). Cells were grown at 37°C in a humidified 5% carbon dioxide atmosphere and were routinely subcultured every 4 days. Cell density and viability were assessed by trypan blue exclusion, using a haemocytometer.

### Fas-induced cell death

Cells were plated in 24-well plates and treatment with anti-Fas IgM (200 ng ml$^{-1}$) was carried out for 24 h. Whenever used, cytotoxic drugs (100 ng ml$^{-1}$ camptothecin, 2.5 µg ml$^{-1}$ anisomycin), were added 5 min prior to anti-Fas IgM. z-VAD was added 5 min prior the addition of cytotoxics.

### Morphological analysis

Cells were harvested and cytospun onto slides. Cells were then stained using the Rapi-Diff II kit (LanganBack, Ireland), which is consists of a fixing solution (100% methanol), an acid dye which stains the nucleus (Eosin Y, 0.1% w/v; formaldehyde phosphate dibasic, 0.4% w/v; potassium phosphate monobasic, 0.5% w/v), and a basic dye which stains the cytoplasm (methylene blue-polychrom, 0.4% w/v; azure A, 0.4% w/v; sodium phosphate dibasic, 0.4% w/v; potassium phosphate monobasic, 0.5% w/v). Cell morphology was assessed by light microscopy, using previously defined criteria, such as membrane blebbing, cell shrinkage and chromatin condensation (Wyllie et al, 1980).

### Western blot

Whole cell lysates were prepared by solubilizing ~10$^6$ cells in 20 µl of RIPA buffer (50 mM Tris–HCl, pH 7.4; 150 mM sodium chloride (NaCl); 1 mM EGTA; 0.25% v/v sodium deoxycholate; 1.0% v/v NP-40; 1 mM sodium orthovanadate; 1 mM sodium fluoride; 0.1 mM phenylmethylsulphonyl fluoride; 1.0 µg ml$^{-1}$ antipain; 1.0 µg ml$^{-1}$ aprotinin; 1.0 µg ml $^{-1}$ chymostatin; 0.1 µg ml$^{-1}$ leupeptin; 4.0 µg ml$^{-1}$ pepstatin) and incubating cells on ice for 1 h. Prior to electrophoresis, cells were diluted in 2 × SDS (sodium dodecyl sulphate) gel-loading buffer (100 mM Tris–HCl, pH 6.8; 200 mM dithiothreitol (DTT); 4.0% w/v SDS; 0.2% w/v bromophenol blue; 20% v/v glycerol) and boiled for 15 min. Proteins were separated in a 10% (w/v) polyacrylamide gel and blotted onto Trans-Blot nitrocellulose membrane. After blocking, the membrane was incubated with the primary antibody of interest (anti-c-Jun, 2.5 µg ml$^{-1}$; anti-SAPK/JNK, 1 µg ml$^{-1}$) for 1 h at room temperature, followed by the relevant secondary horseradish peroxidase (HRP)-conjugated antibody. The membrane was probed with enhanced chemiluminescence (ECL) (Amersham) and exposed to Kodak X-OMAT LS film (Sigma).

### Measurement of SAPK/JNK activity

SAPK/JNK kinase activity was measured using a non-radioactive kit (New England Biolabs) which employs an N-terminal c-Jun fusion protein bound to glutathione sepharose beads to selectively precipitate SAPK/JNK from cell lysates.

#### *Preparation of cell lysates*

DU145 (1×10$^6$) were plated in 6-well plates and cultured for 24 h. Cells were treated with either camptothecin (100 ng ml$^{-1}$), anti-Fas IgM (200 ng ml$^{-1}$), or both. At the appropriate times, after a wash with cold phosphate buffered saline (PBS), 500 µl of 1 × lysis buffer (20 mM Tris, pH 7.4; 150 mM NaCl; 1 mM EGTA; 1 mM EDTA; 1% v/v Triton X-100; 2.5 mM sodium pyrophosphate; 1 mM β-glycerolphosphate; 1 mM $Na_3VO_4$; 1 µg ml$^{-1}$ leupeptin; 1 mM PMSF) was added to cells and the plate was incubated on ice for 5 min. The cells were scraped off the plate, transferred to microcentrifuge tubes and sonicated 4 times for 5 s. Cells were then centrifuged at 14 000 rpm for 10 min at 4°C and the supernatants were stored at –70°C.

#### *SAPK/JNK 'immunoprecipitation'*

GSH-c-Jun fusion protein (2 µg) was added to protein sample (250 µg) and incubated overnight at 4°C. Samples were

 © 2000 Cancer Research Campaign

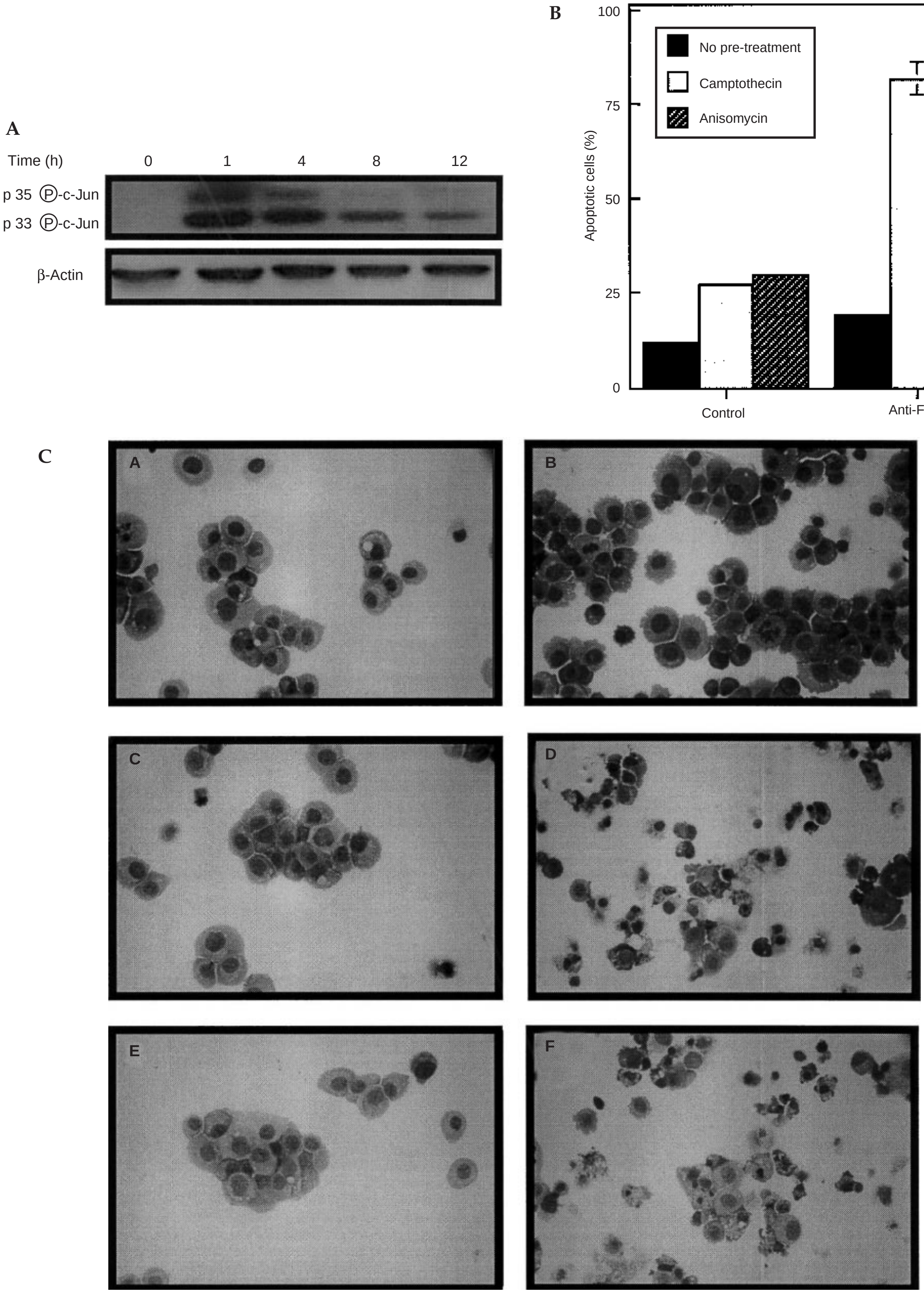

**Figure 1** Anisomycin causes an early and persistent activation of SAPK/JNK, and sensitiszes DU145 cells to anti-Fas in a manner analogous to camptothecin. (**A**) SAPK/JNK activity is evident 1 h following treatment with anisomycin (2.5 µg $ml^{-1}$), and remains elevated at least until 12 h post-treatment. As an additional control for initial protein concentration, β-actin was assessed in supernatant extracts. (**B**) Cells were treated with camptothecin (100 ng $ml^{-1}$), or anisomycin (2.5 µg $ml^{-1}$), and concomitantly incubated with anti-Fas IgM (200 ng $ml^{-1}$), for 24 h, and apoptosis levels were assessed by morphological analysis. The data (from three independent microscopic fields) represents mean apoptosis ± standard error of the mean. (**C**) Typical cellular morphology of (*A*) untreated DU145 cells, and cells treated with (*B*) anti-Fas IgM, (*C*) camptothecin, (*D*) camptothecin plus anti-Fas; (*E*) anisomycin; and (*F*) anisomycin plus anti-Fas. The results shown are representative of at least three independent experiments

© 2000 Cancer Research Campaign 

microcentrifuged at 14 000 rpm for 1 min at 4°C. The pellets were washed twice with 500 μl of 1 × lysis buffer, followed by two washes with 500 μl of 1 × kinase buffer (25 mM Tris, pH 7.5; 5 mM β-glycerolphosphate; 2 mM DTT; 0.1 mM $Na_3VO_4$; 10 mM magnesium chloride ($MgCl_2$)).

### *Kinase assay*

The pellet was then resuspended in 50 μl 1 × kinase buffer supplemented with 240 μM ATP and incubated for 30 min at 30°C. The reaction was terminated with 25 μl 3 × SDS sample buffer (187.5 mM Tris–HCl, pH 6.8; 6% w/v SDS; 30% v/v glycerol; 150 mM DTT, 0.3% w/v bromophenol blue). Samples were boiled for 5 min and then microcentrifuged for 2 min. Samples were then run on a 10% (w/v) SDS polyacrylamide gel.

### *Western immunoblotting and detection of proteins*

The membrane was blocked with blocking buffer (1 × TBS; 0.1% Tween-20; 5% w/v non-fat dry milk) for 1 h at room temperature, and then incubated with a phospho-specific-c-Jun antibody (1/1000 dilution in 1 × TBS, 0.05% v/v Tween-20; 5% w/v BSA) at 4°C, overnight. The membrane was incubated with anti-rabbit-HRP-conjugated antibody (1/2000) for 1 h, at room temperature, and probed with Phototope®-HRP Western blot detection kit (New England BioLabs) before exposure to Kodak X-OMAT LS film (Sigma).

### Inhibition of SAPK/JNK activity by antisense olinucleotides

The antisense (AS) sequences were chosen based on their ability to eliminate steady target RNA > 95% (Bost et al, 1997). Two antisense sequences against the two major forms of SAPK/JNK (JNK1 and JNK2) were chosen with sequences 5′-CT-CTCTGTAGGCCCGC TTGG-3′ and 5′-GTCCGGGCCAGGCC-AAAGTC-3′. The sequences of the control (NS) oligonucleotides were 5′-CTTTCCGTTGGACCCCTGGG-3′ and 5′-GTGCGCG-GAGCCCGAAATC-3′. Cells were harvested by trypsinization, washed twice with warm PBS, then resuspended in electroporation buffer (control samples, PBS only; AS-treated samples, PBS containing 2 μM JNK1 AS and 2 μM JNK2 AS; NS-treated samples, PBS containing 2 μM JNK1 NS and 2 μM JNK2 NS) at $1 \times 10^6$/0.8 ml, and incubated at room temperature for 10 min. The oligonucleotides were delivered by electroporation (0.15 mV, 960 mF) using a Gene Pulser (BioRad). Following incubation on ice for 14 min, cells were washed twice with cold PBS, resuspended in complete media (RPMI-1640; FCS, 5% v/v; L-Gln,\ 2 mM), supplemented with antibiotics. Cells were then plated in 6-well plates and cultured for 24 h, after which period treatments with anti-Fas (200 ng $ml^{-1}$) and camptothecin (100 ng $ml^{-1}$) were carried out. At the appropriate times, protein extracts and cytospins were prepared, and analysed as previously described.

### RESULTS

### Anisomycin potentiates anti-Fas-mediated cytotoxicity in a manner analogous to camptothecin

Anisomycin has previously been shown to act exactly like a signalling agonist in eliciting highly specific and virtually complete homologous desensitization of immediate early (IE) gene induction and stress kinase activation (Hazzalin et al, 1998). Indeed, in DU145 prostate cancer cells anisomycin induces an early and persistent activation of SAPK/JNK (Figure 1A). There is some controversy in the literature as to whether or not SAPK/JNK activity can influence Fas-induced apoptotic signalling pathways. We have recently shown that DU145 cells were highly resistant to anti-Fas IgM treatment. This resistance was overcome by pretreating the cells with sublethal doses of camptothecin. To address the question of whether camptothecin was sensitizing DU145 cells to anti-Fas via a mechanism which involved activation of SAPK/JNK, we elevated the activity of SAPK/JNK using anisomycin and assessed cell sensitivity to Fas ligation. Cells were treated for 24 h with equally sublethal doses of anisomycin (2.5 μg $ml^{-1}$) and anti-Fas (200 ng $ml^{-1}$), and apoptosis levels were compared with those induced by concomitant treatment of cells with camptothecin (100 ng $ml^{-1}$) and anti-Fas IgM. Figure 1B and IC shows that anisomycin potentiated anti-Fas-induced apoptosis to exactly the same extent as camptothecin, suggesting that elevation of SAPK/JNK activity can sensitize DU145 prostate cancer cells to anti-Fas-induced apoptosis. Anisomycin has been reported to activate both SAPK/JNK and p38MAPK. We repeated our Fas sensitization experiments in the presence of a specific p38MAPK inhibitor SB203580 (Juo et al, 1997). Inhibition of this kinase had no effect on sensitization to Fas-mediated apoptosis (data not shown).

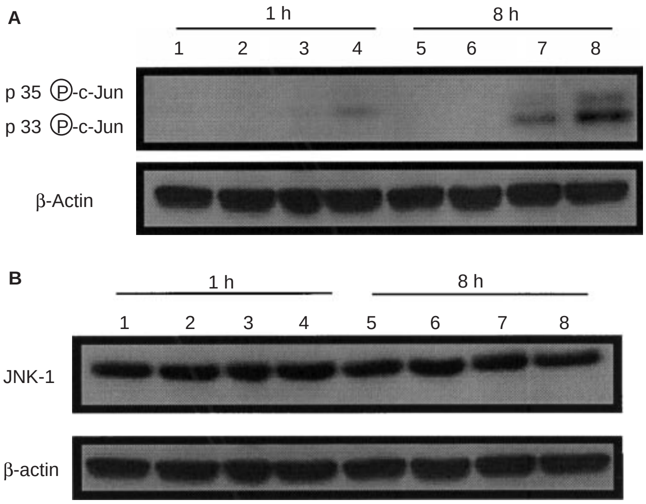

**Figure 2** Camptothecin, but not anti-Fas, strongly and persistently activates SAPK/JNK in DU145 prostate cancer cells. (**A**) SAPK/JNK activity was measured on extracts of untreated cells (lanes 1 and 5), and of cells treated with anti-Fas (200 ng $ml^{-1}$) (lanes 2 and 6), camptothecin (100 ng $ml^{-1}$) (lanes 3 and 7), or both (lanes 4 and 8) for 1 (lanes 1–4), or 8 h (lanes 5–8). SAPK/JNK activity is evident 1 h after treatment with camptothecin and anti-Fas, and it increases in a time-dependent manner. Although anti-Fas fails to activate SAPK/JNK, it potentiates camptothecin-induced SAPK/JNK activity. (**B**) Endogenous expression of SAPK/JNK is not altered by treatment with camptothecin, anti-Fas, or both

### Camptothecin, but not anti-Fas, strongly and persistently activates SAPK/JNK in DU145 cells

To address the question of whether camptothecin was sensitizing the DU145 cells to anti-Fas by activating the SAPK/JNK cascade, cells were treated with anti-Fas (200 ng $ml^{-1}$), camptothecin (100 ng $ml^{-1}$), or both, for varying periods of time, and the kinase

*© 2000 Cancer Research Campaign*

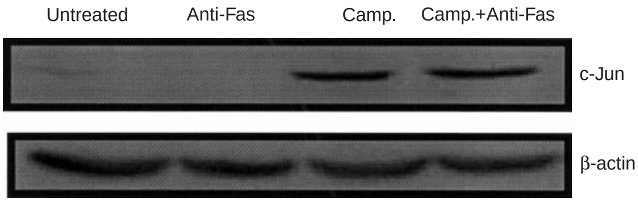

**Figure 3** The c-Jun protein expression in DU145 cells increases after treatment with camptothecin. Protein extracts of untreated cells, and cells treated with anti-Fas (200 ng ml$^{-1}$), camptothecin (100 ng ml$^{-1}$), or camptothecin and anti-Fas, for 24 h, were run on a 10% (w/v) SDS-PAGE. β-actin was also probed for to ensure equal protein loading

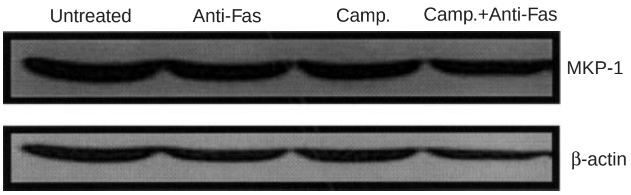

**Figure 4** MKP-1 expression in DU145 cells. Expression was determined in untreated cells, and in cells treated with anti-Fas (200 ng ml$^{-1}$), camptothecin (100 ng ml$^{-1}$), or with both for 24 h. β-actin was probed to ensure equal protein loading of the gel

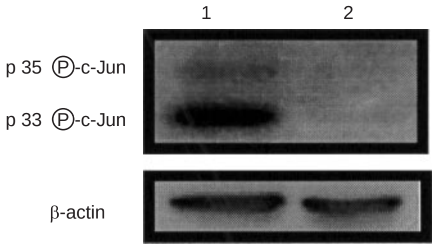

**Figure 5** Inhibition of caspases has no effect on SAPK/JNK activation by camptothecin and anti-Fas. SAPK/JNK activity was measured in untreated (lane 2) DU145 cells, and cells treated with camptothecin (100 ng ml$^{-1}$) and anti-Fas (200 ng ml$^{-1}$) in the presence of z-VAD (50 μM), for 12 h (lane 1). As a control, β-actin was assessed in supernatant fractions activity was measured as described in Materials and Methods. Camptothecin strongly and persistently activated SAPK/JNK (Figure 2A). Anti-Fas treatment alone failed to induce SAPK/JNK, but it potentiated the activation induced by camptothecin. The endogenous levels of SAPK/JNK (Figure 2B) remained unchanged, indicating that camptothecin and anti-Fas have no effect on SAPK/JNK expression levels.

### Expression of c-Jun is induced by camptothecin, but not by anti-Fas c-Jun is an inducible transcription factor which directs changes of gene expression in response to multiple extracellular stimuli. Transcription of *c-jun* mRNA rises after exposure to cytokines and cytotoxic stresses (Ham et al, 1995; Leppä et al, 1998). However, the activity of c-Jun can also be modulated at the protein level. Indeed, a number of regulatory phosphorylations occur on Ser63 and Ser73, and Thr91 and/or Thr93 within the *trans*-activation domain of c-Jun. These phosphorylations result in the stabilization of c-Jun, as well as enhanced *trans*-activation and DNA-binding activity (Smeal et al, 1992; Lëppa et al, 1998). Importantly, c-Jun has been shown to be activated during the initiation of apoptosis induced by radiation and chemotherapy in the prostate (Furuya and Isaacs, 1993). Here, we show that treatment with camptothecin, but not with anti-Fas, induced c-Jun expression (Figure 3), providing further evidence for an important role of SAPK/JNK cascade in camptothecin-mediated anti-Fas-induced apoptosis in prostate cancer cells.

### Expression of MKP-1 remains unchanged following treatment with camptothecin and anti-Fas

The mitogen-activated protein kinase phosphatase 1 (MKP-1) is induced by several oncogenes in the Ras-dependent pathway and it can inactivate both the MAPK (ERK1/ERK2), and the SAPK/JNK pathways (Magi-Galluzzi et al, 1997). In addition, it has been shown that MKP-1 is often overexpressed in prostate cancers and that it inhibits apoptosis in human prostate tumours (Magi-Galluzzi et al, 1997). Moreover, SAPK/JNK enzymatic activity seems to be inversely related to MKP-1 expression (Magi-Galluzzi et al, 1998). To determine whether camptothecin sensitization was mediated via inhibition or down-regulation of MKP-1, cells were treated as described above and the expression of MKP-1 assessed by Western blot analysis (Figure 4). Neither camptothecin, nor anti-Fas had any effect on MKP-1 expression, indicating that camptothecin's effect was independent of MKP-1 expression levels.

### Camptothecin-induced activation of SAPK/JNK is an upstream event in the Fas-signalling pathway

Fas signalling has been shown to involve sequential activation of caspases (Enari et al, 1996; Logthorne et al, 1997). We have previously shown that camptothecin and anti-Fas-induced apoptosis involved caspases, as apoptosis could be blocked by the caspase pan-inhibitor z-VAD (Costa-Pereira and Cotter, 1999). To investigate whether inhibition of caspase activity influenced camptothecin-induced SAPK/JNK activation, cells were treated with camptothecin (100 ng ml$^{-1}$), and anti-Fas (200 ng ml$^{-1}$) in the presence of z-VAD (50 μM) and the activity of SAPK/JNK was determined as previously. Although z-VAD completely blocked anti-Fas-mediated cytotoxicity, it had no effect on camptothecin-induced SAPK/JNK activity (Figure 5). These results suggest that SAPK/JNK activation is not caspase-dependent, and that SAPK/JNK activation is not a secondary effect of the death induced by anti-Fas. Furthermore, the results indicate that camptothecin-induced activation of SAPK/JNK is upstream from caspases and, thus an early event in the Fas-signalling pathway.

### SAPK/JNK activity is absolutely required for the induction of apoptosis in DU145 cells by camptothecin and anti-Fas

Experiments thus far have demonstrated that the induction of stress kinase activity parallels camptothecin sensitization of DU145 cells to anti-Fas-induced apoptosis. Moreover, the induction of SAPK/JNK by anisomycin mimicked the effects mediated by camptothecin. In order to establish whether SAPK/JNK activity is specifically required for the sensitization to Fas-mediated apoptosis we have used phosphorothioate antisense oligonucleotides

*© 2000 Cancer Research Campaign* 

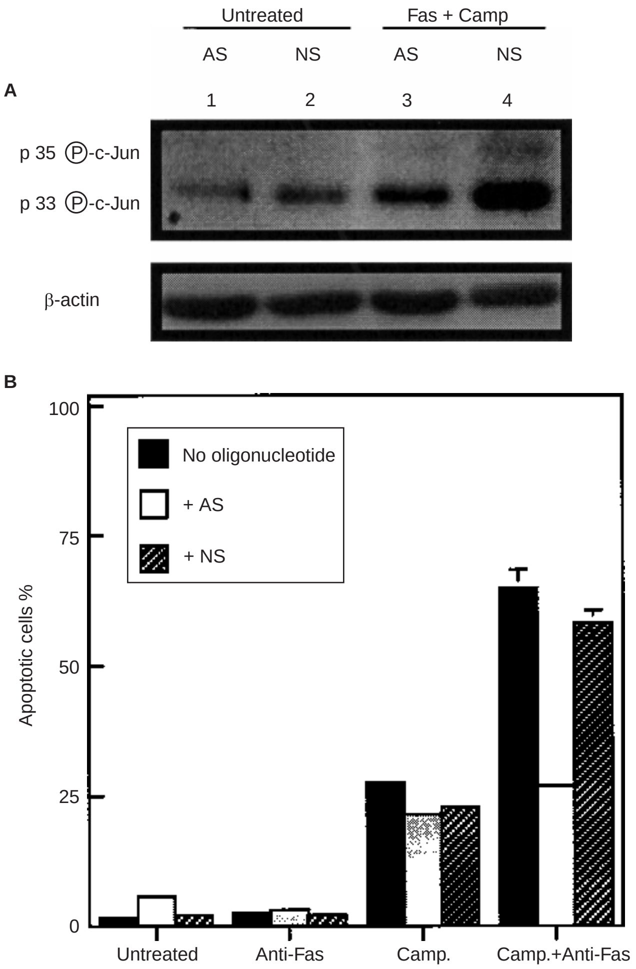

**Figure 6** Inhibition of SAPK/JNK activity by antisense oligonucleotides targeted to JNK1 and JNK2. (**A**) The activity of SAPK/JNK was assessed in cells which had been electroporated in the presence of JNK AS (lanes 1 and 3), or in the presence of JNK NS (lanes 2 and 4). In order to activate SAPK/JNK cells were treated with camptothecin (100 ng $ml^{-1}$) and anti-Fas (200 ng $ml^{-1}$), for 8 h (lanes 3 and 4). As a control, β-actin was assessed in supernatant fractions. (**B**) Inhibition of SAPK/JNK activity by JNK1 and JNK2 antisense oligonucleotides reverses camptothecin sensitization of DU145 cells to anti-Fas-induced apoptosis. Cells were electroporated in the absence of oligonucleotides (■); or in the presence of SAPK/JNK As (■); or SAPK/JNK NS (■), and subsequently treated for 24 h with anti-Fas (200 ng $ml^{-1}$), camptothecin (100 ng $ml^{-1}$), or both. Apoptosis levels were assessed as described in Figure 1A. The data (from three independent microscopic fields) represents mean apoptosis ± standard error of the mean and the results shown are representative of three independent experiments targeted to JNK1 and JNK2 to block the SAPK/JNK pathway. Cells were electroporated in the presence of antisense (AS) oligonucleotides, or non-sense (NS) oligonucleotides, as described in Materials and Methods, and allowed to recover for 24 h. The activity of SAPK/JNK was measured in untreated cells and in cells treated with camptothecin and anti-Fas (8 h). Figure 6A shows that concomitant treatment of DU145 cells with camptothecin, and anti-Fas strongly activates SAPK/JNK in cells treated with NS, but not in cells treated with the AS, indicating that the AS oligonucleotides were causing a specific inhibition of SAPK/JNK activity. A slight induction of SAPK/JNK activity was noted in untreated cells, suggesting that electroporation causes some stress to the cells. This is, however, not comparable to the activation induced by treatment with camptothecin and anti-Fas.

After electroporation in the presence of 2 μM of JNK1 AS and 2 μM of JNK2 AS, cells were allowed to recover for 24 h, and were then treated with anti-Fas (200 ng $ml^{-1}$), camptothecin (100 ng $ml^{-1}$), or both for 24 h. As a control, cells were electroporated in the presence of NS, or in the absence of oligonucleotides. Inhibition of SAPK/JNK by AS oligonucleotides dramatically reduced the apoptosis of DU145 cells induced by concomitant treatment with camptothecin and anti-Fas. Apoptosis levels were comparable to those induced by treatment with camptothecin alone (Figure 6B). Treatment of cells with NS oligonucleotides had no significant effect on camptothecin sensitization of DU145 prostate cancer cells to anti-Fas-induced apoptotic cell death, thus suggesting that inhibition of apoptosis is specific to SAPK/JNK inhibition. These results strongly support a key role for SAPK/JNK in Fas-signalling in DU145 prostate cancer cells.

### DISCUSSION

Although there is still some controversy in the literature about the relevance of SAPK/JNK activation in the cell death pathway(s) induced by Fas, activation of Fas itself has been shown to activate SAPK/JNK even when this activity is not essential for apoptosis (Lenczowski et al, 1997). In this case, cells were sensitive to Fas alone and did not require a secondary signal for sensitization.

DU145 cells express both Fas and FasL, but are resistant to anti-Fas-mediated apoptosis, unless pretreated with sublethal doses of a cytotoxic drug, such as camptothecin (Costa-Pereira and Cotter, 1999). Here, we show that treatment of DU145 prostate cancer cells with anti-Fas failed to activate SAPK/JNK. Concomitant treatment of cells with camptothecin and anti-Fas, however, strongly and persistently activated SAPK/JNK, which correlated well with cell death. In addition, anisomycin, a strong activator of SAPK/JNK, synergized with anti-Fas to the same extent as camptothecin, suggesting that SAPK/JNK activation was indeed required for DU145 cells to undergo anti-Fas-induced apoptosis. Moreover, camptothecin, but not anti-Fas, induced expression of c-Jun, indicating that camptothecin not only modulates the SAPK/JNK cascade by mediating the phosphorylation and activation of SAPK/JNK (which, in turn, will presumably activate c-Jun), but also modulates the pathway at the protein level by inducing c-Jun expression. MKP-1 expression levels have been reported to be elevated in prostate cancer. MKP-1 protein levels remained constant after treatment of cells with camptothecin, suggesting that modulation of SAPK/JNK activity by camptothecin is independent of MKP-1. It remains possible, however, that alterations of MKP-1 activity may be involved in the modulation of SAPK/JNK.

We have previously shown that activated caspases were required for this cytotoxicity (Costa-Pereira and Cotter, 1999). However, inhibition of caspase activity by the pan-inhibitor z-VAD had no effect on SAPK/JNK activation following concomitant treatment of cells with camptothecin and anti-Fas, suggesting that SAPK/JNK activation was not caspase-dependent. The results also indicate that SAPK/JNK activation is not a secondary effect of the death induced by camptothecin and anti-Fas, and that camptothecin-induced activation of SAPK/JNK is thus an upstream and early event, which is important for Fas signalling in these cells.

Antisense oligonucleotides targeted to JNK1 and JNK2 reversed camptothecin's effect(s), indicating that the SAPK/JNK cascade plays a critical role in Fas-mediated apoptotic cell death in DU145 prostate cancer cells. These cells are resistant to apoptosis

 © 2000 Cancer Research Campaign induced by anti-Fas treatment, even at high concentrations (e.g. 1.5 μg $ml^{-1}$, data not shown). The fact that anti-Fas antibodies fail to induce apoptosis in DU145 cells and to activate SAPK/JNK, and the fact that camptothecin, a cytotoxic drug which has been shown to activate SAPK/JNK, sensitizes the cells to Fas-mediated cytotoxicity, together with the AS data, strongly suggest that SAPK/JNK plays an important role in Fas-signalling pathway(s) in these cells.

Other groups have reported that cells which are sensitive to apoptosis, or have seen sensitized to apoptosis, can activate stress kinases downstream of caspases (Juo et al, 1998). MEKK1 has been shown to be cleaved and activated by a caspase, which may then be responsible for SAPK/JNK activation (Xu and Cobb, 1997). In our system we have evidence of this also, as in cells which had been induced to undergo apoptosis (Fas and camptothecin), SAPK/JNK activation was higher than in cells which were alive but had elevated SAPK/JNK activity due to the presence of camptothecin alone. Our data, however, demonstrates that in Fas-resistant cell line SAPK/JNK plays a more important role in the upstream regulation of Fas sensitivity, as its activation was required for sensitization to Fas, and was upstream of caspase activation. Another report regarding Fas-insensitive cell lines also indicated the presence of an inhibitor upstream of caspases (Rokhlin et al, 1998). It is notable that the TRAIL/Apo-2 ligand can also activate SAPK/JNK by both caspase-dependent and -independent pathways (Muhlenbeck et al, 1998).

The nature by which SAPK/JNK sensitizes cells to Fas remains to be established. Frost and colleagues (1999) have suggested that drug-mediated sensitization to Fas killing may be due to alterations in pre-existing proteins, and that this phenomena is independent of de novo protein synthesis. Activation of a kinase such as SAPK/JNK by cytotoxic drugs may lead to phosphorylation and activation of activator proteins and/or inhibition of inhibitory proteins, independently of Fas and of caspases, in parallel signalling cascades. This would presumably release the inhibition in Fas-mediated apoptosis signalling pathways.

Fas can activate at least two independent pathways: the Fas-FADD-caspase 8 axis, which leads to a caspase cascade and apoptosis, and the more recently described Fas-Daax-ASK1 axis, which leads to the activation of SAPK/JNK and/or p38MAPK cascades (Chang et al, 1998). Chang and colleagues (1998) provide evidence for a critical role of ASK1 in SAPK/JNK activation and apoptosis induced by Fas binding of Daax, and suggest that the Daax-ASK1 axis provides a mechanism for caspase-independent activation of SAPK/JNK by Fas and other stimuli. Thus, the inhibition in Fas signalling could also be at the level of Daax-ASK1.

Bcl-2 is often over-expressed in prostatic cancers (Krajewska et al, 1996), and it is possible that these proteins play a role in the loss of sensitivity to Fas-mediated apoptosis in the prostate. Interestingly, it has recently been shown that overexpression of SAPK/JNK antagonized the anti-apoptotic effect of Bcl-2, presumably due to sustained activation of SAPK/JNK (Park et al, 1997). Pro-apoptotic Bcl-2 family members, such as Bax, can antagonize the effects exerted by the anti-apoptotic members. Indeed, it is believed that the ratios between these proteins are important determinants of the cell's fate following injury (Reed, 1997). Phosphorylation of Bcl-2 has been shown to inactivate its anti-apoptotic function by displacing it from Bax, thereby altering the ratios between these two proteins (Haldar et al, 1996). Importantly, Maundrell and colleagues (1997) have shown that Bcl-2 could be phosphorylated by SAPK/JNK, and Yamamoto et al (1999) have more recently shown that Bcl-2 is phosphorylated by a cascade involving ASK1 which has also been implicated in Fas signalling (Chang et al, 1998). Persistent activation of SAPK/JNK by camptothecin may, therefore, induce apoptosis by eliciting different, but complementary, cellular responses. It remains possible that activation of SAPK/JNK by camptothecin may induce, via c-Jun or other transcription factors, the transcription of genes important for apoptosis. More likely, however, is the possibility that SAPK/JNK may directly phosphorylate existing proteins which regulate cell fate, such as Bcl-2, or Bcl-$x_L$, or indeed unidentified proteins.

Interestingly, MKK4/SEK1 has been identified as a candidate tumour suppressor gene (Teng et al, 1997; Su et al, 1998) and more recently as a candidate prostate cancer metastasis suppressor gene encoded by human chromosome 17 (Yoshida et al, 1999). Loss of tumour suppressor and/or metastasis suppressor genes are important events in the progression towards a malignant phenotype. It is tempting to speculate that, by activating SAPK/JNK, camptothecin is mimicking a cellular defence mechanism, which has been lost during tumour progression.

We have characterized here a pathway which may enable prostate cancer cells to escape immunological Fas-induced death. Importantly, SAPK/JNK has been shown to act upstream in the Fas-signalling pathway(s). If loss of sensitivity to Fas signalling is shown to play a direct role in the progression of prostate cancer, then restoring the cells' susceptibility to Fas ligation will be of obvious importance for future medical intervention in prostate cancer patients. These results may, thus, provide us with clues for a better understanding of prostate cancer progression, and for the development of new strategies for the management and treatment of the disease.

### ACKNOWLEDGEMENTS

This work has been supported by grant PRAXIS XXI/BD/5857/95 from the Foundation for Science and Technology (Fundação para a Ciência e a Tecnologia), Lisbon, Portugal and the Health Research Board, Ireland. The authors would like to thank Emma Creagh for many helpful discussions.

### REFERENCES

Bost F, McKay R, Dean N and Mercola D (1997) The JUN kinase/stress-activated protein kinase pathway is required for epidermal growth factor stimulation of growth of human A549 lung carcinoma cells. *J Biol Chem* **272:** 33422–33429

Chang HY, Nishitoh H, Yang X, Ichijo H and Baltimore D (1998) Activation of apoptosis signal-regulating kinase 1 (ASK1) by the adapter protein Daax. *Science* **281**: 1860–1863

Costa-Pereira AP and Cotter TG (1999) Camptothecin sensitises androgen-independent prostate cancer cells to anti-Fas-induced apoptosis. *Br J Cancer* **80**: 371–378

de Souza PL, Cooper MR, Imondi AR and Myers CE (1997) 9-Aminocamptothecin: a topoisomerase I inhibitor with preclinical activity in prostate cancer. *Clin Cancer Res* **3**: 287–294

Dhanasekaran N and Reddy EP (1998) Signalling by dual specificity kinases. *Oncogene* **17**: 1447–1455

Enari M, Talanian RV, Wong WW and Nagata S (1996) Sequential activation of ICE-like and CPP32-like proteases during Fas-mediated apoptosis. *Nature (Lond)* **380**: 723–726

Finch A, Holland P, Cooper J, Saklatavata J and Kracht M (1997) Selective activation of JNK/SAPK by interleukin-1 in rabbit liver is mediated by MKK7. *FEBS Lett* **418**: 144–148

Frost PJ, Belldegrun A and Bonavida B (1999) Sensitisation of immunoresistant prostate carcinoma cell lines to Fas/Fas ligand-mediated killing by cytotoxic lymphocytes: independence of de novo protein synthesis. *Prostate* **41**: 20–30

© 2000 Cancer Research Campaign 

Furuya Y and Isaacs JT (1993) Differential gene regulation during programmed cell death (apoptosis) versus proliferation of prostatic glandular cells induced by androgen manipulation. *Endocrinology* **133**: 2660–2666

Goillot E, Raingeaud J, Ranger A, Tepper RI, Davis RJ, Harlow and Sanchez I (1997) Mitogen-activated protein kinase-mediated Fas apoptotic signalling pathway. *Proc Natl Acad Sci USA* **94**: 3302–3307

Griffith TS, Brunner T, Fletcher SM, Green DR and Ferguson TA (1995) Fas-ligand-induced apoptosis as a mechanism of immune privilege. *Science* **270**: 1189–1192

Gupta S, Campbell D, Derijard B and Davis R (1995) Transcription factor ATF2 regulation by the JNK signal transduction pathway. *Science* **267**: 389–393

Haldar S, Chintapalli J and Croce CM (1996) Taxol induces *bcl-2* phosphorylation and death of prostate cancer cells. *Cancer Res* **56**: 1253–1255

Ham J, Babij C, Whitfield J, Pfarr CM, Lallemand D, Yaniv M and Rubin LL (1995) A c-jun dominant negative mutant protects sympathetic neurons against programmed cell death. *Neuron* **14**: 927–939

Hazzalin CA, Le Panse R, Cano E and Mahadevan LC (1998) Anisomycin selectively desensitises signalling components involved in stress kinase activation and *fos* and *jun* induction. *Mol Cel Biol* **18**: 1844–1854

Hedlund TE, Duke RC, Schleicher MS and Miller GJ (1998) Fas-mediated apoptosis in seven human prostate cancer cell lines: correlation with tumour stage. *Prostate* **36**: 92–101

Hsiang YH, Likan MG and Liu LF (1989) Arrest of replication forks by drug stabilised topoisomerase-I-DNA cleavable complexes as a mechanism of cell killing by camptothecin. *Cancer Res* **49**: 5077–5082

Juo P, Kuo CJ, Reynolds SE, Konz RF, Raingeaud J, Davis RJ, Biemann H-P and Blenis J (1997) Fas activation of the p38 mitogen-activated protein kinase signalling pathway requires ICE/CED-3 family proteases. *Mol Cel Biol* **17**: 24–35

Juo P, Kuo CJ, Yuan J and Blenis J (1998) Essential requirement for caspase-8/FLICE in the initiation of the Fas-induced apoptotic cascade. *Curr Biol* **8**: 1001–1008

Kastan MB, Onyyekwere O, Sidransky D, Vogeltein B and Craig RW (1991) Participation of p53 protein in the cellular response to DNA damage. *Cancer Res* **51**: 6304–6311

Krajewska M, Krajewski S, Epstein JI, Shabaik A, Sauvageot J, Song K, Kitada S and Reed JC (1996) Immunohistochemical analysis of *bcl-2*, *bax*, *bcl-X* and *mcl-1* expression in prostate cancers. *Am J Pathol* **148**: 1567–1576

Kuerbitz SJ, Plunkett BS, Walsh WV and Kastan MB (1992) Wild-type p53 is a cell cycle checkpoint determinant following irradiation. *Proc Natl Acad Sci USA* **89**: 7491–7495

Kyriakis JM, Benerjee P, Nikolakaki E, Dai T, Rubic EA, Amhad MF, Avruch J and Woogett JR (1994) The stress-activated protein kinase subfamily of c-Jun kinases. *Nature (Lond)* **369**: 156–160

Leibson PJ (1997) The Fas pathway in apoptosis. *Adv Pharmacol* **41**: 107–132

Leppä S, Saffrich R, Ansorge W and Bohmann D (1998) Differential regulation of c-Jun by ERK and JNK during PC12 differentiation. *EMBO J* **17**: 4404–4413

Lenczowski JM, Dominguez L, Eder AM, King LB, Zacharchuk CM and Ashwell JD (1997) Lack of a role for jun kinase and AP-1 in Fas-induced apoptosis. *Mol Cel Biol* **17**: 170–181

Longthorne VL and Williams GT (1997) Caspase activity is required for commitment to Fas-mediated apoptosis. *EMBO J* **16**: 3805–3812

Magi-Galluzzi C, Mishra R, Fiorentino M, Montironi R, Yao H, Capodieci P, Wishnow K, Kaplan I, Stork PJS and Loda M (1997) Mitogen-activated protein kinase phosphatase 1 is overexpressed in prostate cancers and is inversely related to apoptosis. *Lab Invest* **76**: 37–51

Magi-Galluzzi C, Montironi R, Yao H, Cangi MG, Wishnow K and Loda M (1998) Mitogen-activated protein kinases and apoptosis in PIN. *Virchows Arch* **432**: 407–413

Maundrell K, Antonsson B, Magnenat E, Camps M, Muda M, Chabert C, Gillieron C, Boschert U, Vial-Knecht, Martinou J-C and Arkinstall S (1997) Bcl-2 undergoes phosphorylation by the c-Jun N-terminal kinase/stress-activated protein kinases in the presence of the constitutively active GTP-binding protein Rac 1. *J Biol Chem* **272**: 25238–25242

Muhlenbeck F, Haas E, Schwenzer R, Schubert G, Grell M, Smith C, Scheurich P and Wajant H. TRAIL/Apo2L activates c-Jun NH2-terminal kinase (JNK) via caspase-dependent and caspase-independent pathways. *J Biol Chem* **273**: 33091–33098

O'Connell J, O'Sullivan GC, Collins JK and Shanahan F (1996) The Fas counterattack: Fas-mediated cell killing by colon cancer cells expressing Fas ligand. *J Exp Med* **184**: 1075–1082

Oehm A, Berhmann I, Falk W, Pawlita M, Maier G, Klas G, Li-Weber CM, Richards S, Dhein J, Trauth BC, Ponsting H and Krammer PH (1992) Purification and molecular cloning of the APO-1 cell surface antigen, a member of the tumour necrosis factor/nerve growth factor receptor superfamily: sequence identity with the Fas antigen. *J Biol Chem* **267**: 10709–10715

Park J, Kim I, Oh YJ, Lee K-W, Han P-L and Choi E-J (1997) Activation of c-Jun N-terminal kinase antagonises an anti-apoptotic action of Bcl-2. *J Biol Chem* **272**: 16725–16728

Reed JC (1997) Bcl-2 family proteins: role in dysregulation of apoptosis and chemoresistance in cancer. In: *Apoptosis and Cancer*, Martin SJ (ed). Karger Landes Systems, Basel, Switzerland.

Rokhlin OW, Glover RA and Cohen MB (1998) Fas-mediated apoptosis in human prostatic carcinoma cell lines occurs via activation of caspase-8 and caspase-7. *Cancer Res* **58**: 5870–5875

Sánchez-Perez I, Murguía JR and Perona R (1998) Cisplatin induces a persistent activation of JNK that is related to cell death. *Oncogene* **16**: 533–540

Smeal T, Binetruy B, Mercola DA, Birrer M and Karin M (1991) Oncogenic and transcriptional cooperation with Ha-Ras requires phosphorylation of c-Jun on serines 63 and 73. *Nature (Lond)* **354**: 494–496

Su GH, Hilgers W, Shekher MC, Tang DJ, Yeo CJ, Hruban RH and Kern SE. Alterations in pancreatic, biliary, and breast carcinomas support MKK4 as a genetically targeted tumour suppressor gene. *Cancer Res* **58**: 2339–2342

Teng DH, Perry WL, Hogan JK, Baumgard M, Bell R, Berry S, Davis T, Frank D, Frye C, Hattier T, Hu R, Jammulapati S, Janecki T, Leavitt A, Mitchell JT, Pero R, Sexton D, Schroeder M, Su PH, Swedlund B, Kyriakis JM, Avruch J, Bartel P, Wong AK, Oliphant A, Thomas A, Skolnick MH and Tavtigan SV (1997) Human mitogen-activated protein kinase kinase 4 as a candidate tumour suppressor. *Cancer Res* **57**: 4177–4182

Toyashima F, Moriguchi T and Nishida E (1997) Fas induces cytoplasmic apoptotic responses and activation of the MKK7-JNK/SAPK and MKK6-p38 pathways independent of CPP32-like proteases. *J Cell Biol* **139**: 1005–1015

Uslu R, Borsellino N, Frost P, Gában H, Ng C-P, Mizutani Y, Belldegrun A and Bonavida B (1997) Chemosensitization of human prostate carcinoma cell lines to anti-Fas-mediated cytotoxicity and apoptosis. *Clin Cancer Res* **3**: 963–972

Villunger A, Egle A, Marschitz I, Kos M, Böck G, Ludwig H, Geley S, Kofler R and Greil R (1997) Constitutive expression of Fas (APO-1/CD95) ligand on multiple myeloma cells: a potential mechanism of tumour-induced suppression of immune surveillance. *Blood* **90**: 12–20

Wyllie AH, Kerr JFR and Currie AR (1980) Cell death: the significance of apoptosis. *Int Rev Cytol* **68**: 251–306

Xu S and Cobb MH (1997) MEKK1 binds directly to the c-Jun N-terminal kinases/stress-activated protein kinases. *J Biol Chem* **272**: 32056–32060

Yamamoto K, Ichijo H and Korsmeyer SJ (1999) BCL-2 is phosphorylated and inactivated by an ASK1/Jun N-Terminal protein kinase pathway normally activated at G2/M. *Mol Cel Biol* **19**: 8469–8478

Yonehara S, Ishi A and Yonehara M (1989). A cell-killing monoclonal antibody (anti-Fas) to a surface antigen co-downregulated with the receptor of tumour necrosis factor. *J Exp Med* **169**: 1747–1756

Yoshida BA, Dubauskas Z, Chekmareva MA, Christiano TR, Stadler WM and Rinker-Schaeffer CW (1999) Mitogen-activated protein kinase kinase 4/Stress-activated protein/Erk kinase 1 (*MKK4/SEK1*), a prostate cancer metastasis suppressor gene encoded by human chromosome 17. *Cancer Res* **59**: 5483–5487

*© 2000 Cancer Research Campaign*
